# Correspondence

**Published:** 1901-10

**Authors:** J. P. Foster

**Affiliations:** Selby, S. D.


### CORRESPONDENCE.

SELBY, S. D., August 26, 1901.

EDITOR JOURNAL OF COMPARATIVE MEDICINE AND VETERINARY ARCHIVES.

DEAR SIR: In the August number of the JOURNAL "your correspondent, James Law" (I follow the gentleman's style of address), replies to my letter which appeared in the July issue of the JOURNAL. If those readers of the JOURNAL who are interested in this discussion (I expect that many of them are thoroughly disgusted with it by this time) care to go to the trouble of carefully reviewing this controversy, which began with my original letter published in the JOURNAL of the March issue and has continued in the issues of April, June, July, and August, I am sure that all fair-minded readers, no matter from what college they may hold their diplomas, will admit that I have conducted this controversy with absolute fairness and with a due regard for the truth (notwithstanding the assertions of "James Law" and Dr. Howe). Professor Law entered into this discussion—probably through a misinterpretation of my meaning—when I stated in my first article that certain Ontario graduates had taken a third year in some of the three-year schools, and "they often at graduation capture first prize over those more fortunate students," etc. I think that I have shown in my letter published in the JOURNAL for July *just what I meant so far as Cornell is concerned*, and in the same letter admitted just what James Law contends with regard to three-year schools. He seems bound to put me on record as being opposed to "higher education," in spite of all I can say. James Law admits, and uses for an argument for his side of the question, that certain mentioned Ontario men that he has "made over" in one session at Cornell were graduates or had "received excellent educations" at certain universities, normal and high schools. That's just where the "shoe pinches." If these men had never attended any other school but Ontario, you and others of your stamp would refer to them as being "poorly educated" or "illy equipped for the practice of their profession" because "they graduated from schools which could grant diplomas on short notice, easy terms," etc. Now, J. Law, I don't say that *you* have made these remarks; but I think that your sympathies are with a certain individual not a hundred miles away from you who has made them.

Just what his name is or how many years of his three-year veterinary course were gained in an agricultural college I will leave you to guess.

Now, if you desire to be fair in the future, J. Law, before condemning an Ontario graduate, either by word or thought, you will first ascertain whether or not he has ever graduated from or attended a normal or a high school; and if he has attended or graduated from either you will not proceed to slander him. It is just possible, J. Law, that among the graduates of the Ontario Veterinary College "there are others" beside those you mention whose education was not limited to "readin', 'ritin', and 'rithmetic" at the time they entered Ontario; and I know that one of your age and wide experience must have heard the well-known axiom, "it's a poor rule that won't work both ways."

J. Law has kindly furnished us with a list of the titles held by professors in the different agricultural colleges and experiment stations, and says that I regret (don't know when I expressed any "regret") the lack of statistics relating to the "incumbents of these positions. He says "we have a showing of two with (apparently) V.S. alone as compared with thirty-eight whose titles indicate that they had the privilege of a fuller education."

It is indeed surprising that he should condescend to create the impression that the titles of D.V.S., D.V.M., M.D.C.—yes, or M.R.C.V.S. or F.R.C.V.S.—indicate that the holders of these degrees have necessarily put in more than two years in a veterinary college. How many years did *you* spend in the Dick College, J. Law (where you got nearly one-fifth of the alphabet hung onto your name); or, in other words, what was the length of the course of your alma mater at the time of your graduation? I take it that you got through without being "plucked," because you have given us to understand that you were a good student and got a "silver medal."

J. Law is either—to use his own expression applied to myself in the April number of the JOURNAL—guilty "of stating an apparent truth in such a way as to convey an entirely false impression to the reader," or else he is densely ignorant in regard to the prescribed courses of certain veterinary colleges in years past. Is it possible that J. Law does not know that the degree of D.V.S. was conferred by the following colleges previous to their becoming three-year schools, viz., American Veterinary College, Kansas City Veterinary College, National Veterinary College (before its affiliation with Columbian University), and Chicago Veterinary

College (previous to 1893)? The graduates of the Chicago Veterinary College from about 1893 until 1897 (the class of 1897 was the first to graduate after the extension of the course to three years) received the degree of M.D.C. The Veterinary Department of the Iowa State College conferred the degree of D.V.M. previous to the time its course was extended to three years (January, 1888), and I am suspicious that J. Law is referring to some of these two-year graduates of Ames (one of whom had a B.S. degree) in the misleading list that he presented to the JOURNAL readers. J. Law has been active in Association matters for many years, and it will be hard work for him to plead ignorance of the conditions I have mentioned with regard to the time required to graduate from these schools in past years, and I can only conclude that it was not ignorance, but *something else*, that prompted him to resort to this ridiculous list, in which he pretends that V.S. represents the lowest standard. How about the three D.V.S.'s and the sixteen D.V.M.'s mentioned in your list, J. Law? Are you *sure* that each one of them had more than two years' veterinary education gained in a veterinary college? J. Law says "thirty-eight, whose titles indicate that they had the privilege of a fuller education." Nineteen of these thirty-eight have degrees of plain D.V.S. and D.V.M. It was possible to receive either of these degrees formerly after completing a two-year course; in which event, how do these "titles indicate that they had the privilege of a fuller education" than the title of plain V.S.?

I might further inform J. Law that the degree of V.S. has been conferred upon the graduates of three-year colleges in the United States and Canada. The earlier graduates of the Veterinary Department of McGill University (at that time the Montreal Veterinary College) received the degree of V.S., as did the graduates of the New York College of Veterinary Surgeons before and after it became a three-year school and up to the time it was consolidated with the American Veterinary College. Therefore, it would seem that J. Law's contention in regard to what the title of V.S. "indicates" amounts to nothing. J. Law says "in quoting Fisher he condemns his whole system." Pray, what is my *system*, J. Law?

I wish to thank those readers of the JOURNAL who have taken the time to "wade through" these tiresome communications of mine, and will further state that I have no desire to longer argue this subject with one so manifestly unfair as J. Law has shown himself to be. He is bound to array me against the cause of

"higher veterinary education," and instead of answering my arguments (which he cannot do, I admit) he goes on in a roundabout way, with a profuse play of words, and twists and distorts my ideas of veterinary education to suit himself.

I am not in the position of the character known as "Cy Prime," in the play of "The Old Homestead," who, after each of his ridiculous statements, would proudly declare that "he could prove it, too, if old Bill Jones was alive." I *think* that I can prove every statement I have made during this controversy (without the assistance of "Bill Jones"). Can you do the same, Professor James Law, F.R.C.V.S. (Edinburgh)? My opponent in this controversy admits in the first paragraph of his communication in the August JOURNAL that I agree with him in regard to the superiority of three-year schools over two-year schools. (Then, for goodness' sake, J. Law, don't again mistake me on this subject.) My only desire at the time I sent in my first communication on this subject was to show the absurdity of two-year graduates of present three-year schools in "pounding" Ontario graduates *as a whole.* Good schools occasionally graduate poor men, and it will not do to condemn a good man in the profession, even though he has not had the advantages of a three-year course. I often wonder why it is that Ontario graduates can calmly sit through a State or National Association meeting and hear themselves insulted without a word of protest. I hope that this controversy is about to be closed, as I am tired of the tactics employed by my opponents; however, if they desire to further carry on this discussion (and think that the readers of the JOURNAL can stand some more of it) I will try and keep up my end, and think that perhaps by the time it comes around for me to again reply to any of their communications I can give the readers of the JOURNAL some information that may prove of interest to them, if not particularly interesting to some of the said opponents. Respectfully,

J. P. FOSTER.

---

FIRST EGYPTIAN CONGRESS OF MEDICINE. The first Egyptian Congress of Medicine will be held at Cairo, Egypt, from the 10th to the 14th of December, 1902. The preliminary programme offers many attractive subjects of general medical interest, notably, "The Medicine of Egypt and the Arabs," by Dr. Eïd; "The Medicine of Ethiopia," by Dr. N. Parissis, and "Tuberculosis in Egypt," by Dr. Ibrahim Pacha Hassan. Address: Dr. Voronoff, Secretary-General, Cairo, Egypt.

